# White matter pathology in alzheimer’s transgenic mice with chronic exposure to low-level ambient fine particulate matter

**DOI:** 10.1186/s12989-022-00485-8

**Published:** 2022-06-30

**Authors:** Ta-Fu Chen, Sheng-Han Lee, Wan-Ru Zheng, Ching-Chou Hsu, Kuan-Hung Cho, Li-Wei Kuo, Charles C.-K. Chou, Ming-Jang Chiu, Boon Lead Tee, Tsun-Jen Cheng

**Affiliations:** 1 Department of Neurology, National Taiwan University Hospital, Taipei, Taiwan; 2 Institute of Environmental and Occupational Health Sciences, College of Public Health, National Taiwan University, Room 720, No. 17, Xuzhou Rd, Taipei, 100 Taiwan; 3 Department of Public Health, College of Public Health, National Taiwan University, Taipei, Taiwan; 4 Institute of Biomedical Engineering and Nanomedicine, National Health Research Institutes, Miaoli, Taiwan; 5 Institute of Medical Device and Imaging, National Taiwan University College of Medicine, Taipei, Taiwan; 6 Research Center for Environmental Changes, Academia Sinica, Taipei, Taiwan; 7 Department of Neurology, Memory and Aging Center, University of California at San Francisco, San Francisco, CA USA

**Keywords:** Ambient air pollution, Alzheimer’s disease, White matter, Optic tract

## Abstract

**Background:**

Air pollution, especially fine particulate matter (PM), can cause brain damage, cognitive decline, and an increased risk of neurodegenerative disease, especially alzheimer’s disease (AD). Typical pathological findings of amyloid and tau protein accumulation have been detected in the brain after exposure in animal studies. However, these observations were based on high levels of PM exposure, which were far from the WHO guidelines and those present in our environment. In addition, white matter involvement by air pollution has been less reported. Thus, this experiment was designed to simulate the true human world and to discuss the possible white matter pathology caused by air pollution.

**Results:**

6 month-old female 3xTg-AD mice were divided into exposure and control groups and housed in the Taipei Air Pollutant Exposure System (TAPES) for 5 months. The mice were subjected to the Morris water maze test after exposure and were then sacrificed with brain dissection for further analyses. The mean mass concentration of PM_2.5_ during the exposure period was 13.85 μg/m^3^. After exposure, there was no difference in spatial learning function between the two groups, but there was significant decay of memory in the exposure group. Significantly decreased total brain volume and more neuronal death in the cerebral and entorhinal cortex and demyelination of the corpus callosum were noted by histopathological staining after exposure. However, there was no difference in the accumulation of amyloid or tau on immunohistochemistry staining. For the protein analysis, amyloid was detected at significantly higher levels in the cerebral cortex, with lower expression of myelin basic protein in the white matter. A diffuse tensor image study also revealed insults in multiple white matter tracts, including the optic tract.

**Conclusions:**

In conclusion, this pilot study showed that even chronic exposure to low PM_2.5_ concentrations still caused brain damage, such as gross brain atrophy, cortical neuron damage, and multiple white matter tract damage. Typical amyloid cascade pathology did not appear prominently in the vulnerable brain region after exposure. These findings imply that multiple pathogenic pathways induce brain injury by air pollution, and the optic nerve may be another direct invasion route in addition to olfactory nerve.

**Supplementary Information:**

The online version contains supplementary material available at 10.1186/s12989-022-00485-8.

## Introduction

Air pollution, which describes substances that exist in the atmosphere that are harmful to living beings, has become a current and growing health problem in the modern industrial world. Air pollution has a complicated composition and can include particulate matter (PM), carbon monoxide, lead, nitrogen dioxide, ozone, and sulfur dioxide. Among them, small PM with a diameter less than 2.5 µm (PM_2.5_) is regarded as the most harmful [1]. The Global Burden of Diseases, Injuries, and Risk Factors Study 2015 (GBD 2015) estimated that PM_2.5_ was the fifth-ranking mortality risk factor and responsible for 7.6% of total global deaths [2]. The association of air pollution with damage in the respiratory or cardiovascular systems has been well documented in animal and epidemiological studies [3–8]. However, an increasing number of publications have also mentioned cognitive decline and dementia related to air pollution [9, 10].

PM exposure can result in brain damage, including decreased brain volume, cognitive decline, abnormal blood brain barrier, and brain neuronal inflammation; in addition, it can also increase the risk of Alzheimer's disease (AD), Parkinson's disease, and even ischemic cerebrovascular disease [11–13]. AD is the most common type of dementia and causes 60–70% of cases. In a meta-analysis and paper review, the overall odds ratio (OR) of air pollutants with AD was 1.32 (95% CI: 1.09–1.61), especially PM_2.5_ [12]. Air pollutants have also been listed as one of the major preventable factors in the 2020 report of the Lancet Commission [14].

The amyloid cascade theory has been postulated to explain the pathogenesis of AD. Extracellular senile plaques and intracellular neurofibrillary tangles accumulating from pathological amyloid β-peptide (Aβ) and hyperphosphorylated tau (P-tau) proteins are thought to be pathognomonic markers of this disease [15]. Elderly individuals with cognitive impairment exposed to higher concentrations of PM_2.5_ possessed more brain Aβ plaques, as revealed on fluorine 18 [18^F^]-labeled florbetapir PET scans [16]. The characteristic fluid markers of AD, decreased Aβ42 and increased P-tau protein levels in cerebrospinal fluid (CSF), were also reported in urbanites with high exposure to PM_2.5_ [11]. Accelerated amyloid plaque deposition and P-tau accumulation have been detected in the brains of animal models after exposure to concentrated air pollutants, especially in the hippocampal area [17–21]. The successive pathways of inflammation, oxidative stress, microglial activation, and even DNA damage are thought to cause this degenerative pathology [17, 19, 22–24].

White matter hyperintensities have traditionally been viewed as a marker of vascular disease, but they were also commonly found in AD pathology in a recent study [25]. However, there was no correlation with the topography of Aβ or tau accumulation, and this pathological change was thought to be attributed to the coexisting vascular processes or disruption of myelin by activated oligodendrocytes [26, 27]. White matter involvement by air pollution exposure has also been reported, but only in a few animal and human studies with high concentrations [28, 29]. However, the exact mechanism still needs to be clarified.

Numerous animal studies have revealed AD-related neurotoxicity in the gray and white matter areas of mouse brains under exposure to high concentrations of PM [17, 23, 30, 31]. However, in the real world, the PM_2.5_ concentration is generally not as high as those in most animal studies discussed above. When simulating the true condition, chronic exposure to ambient air pollution is the best model. In our previous study, AD transgenic mice exposed to ambient air pollution for 3 months only presented minimal AD-like pathology in some gray matter areas, especially the entorhinal cortex [32]. Therefore, we designed this animal study with a longer exposure time, investigated AD-associated pathology and related markers of intermediate pathogenic processes, and studied white matter insult with the diffusion tensor imaging technique of magnetic resonance imaging (DTI-MRI).

## Method

### Taipei air pollutant exposure system (TAPES)

A customized air pollutant exposure system was utilized in this study (see supplement). This system was set in the laboratory of the College of Public Health, National Taiwan University (Taipei, Taiwan). Environmental ambient air was directly introduced into the experimental chambers to mimic real-world conditions. The control group chamber was connected with a high-efficiency particulate air filter. We used 37 mm Teflon filters (Pall Corporation, Port Washington, New York, USA) and a DustTrak™ II Aerosol Monitor 8530 (TSI, Shoreview, Minnesota, USA) to periodically record the mass concentrations and particle size. The detailed design of the structures and materials of this real-world air pollution exposure system has been described in a previous publication [33]. Ion chromatography and inductively coupled plasma mass spectrometry were selected for further analysis of the components of PM_2.5_ [34, 35].

### Animal and experimental protocols

Acclimated and inbred AD transgenic mice (3xTg-AD) were obtained from Prof. Chiu’s team. These mice were housed in individual ventilated cages with a 12-h light/dark cycle, constant temperature (22 ± 2 °C) and humidity (50 ± 5%), and access to diet (LabDiet® 5001; PMI® Nutrition International, Brentwood, MO, USA) and water ad libitum. All management was in accordance with the ethical rules of the Institutional Animal Care and Use Committee of the College of Medicine and the College of Public Health, National Taiwan University (Permit Number: 20160545).

**Fig. 1:**
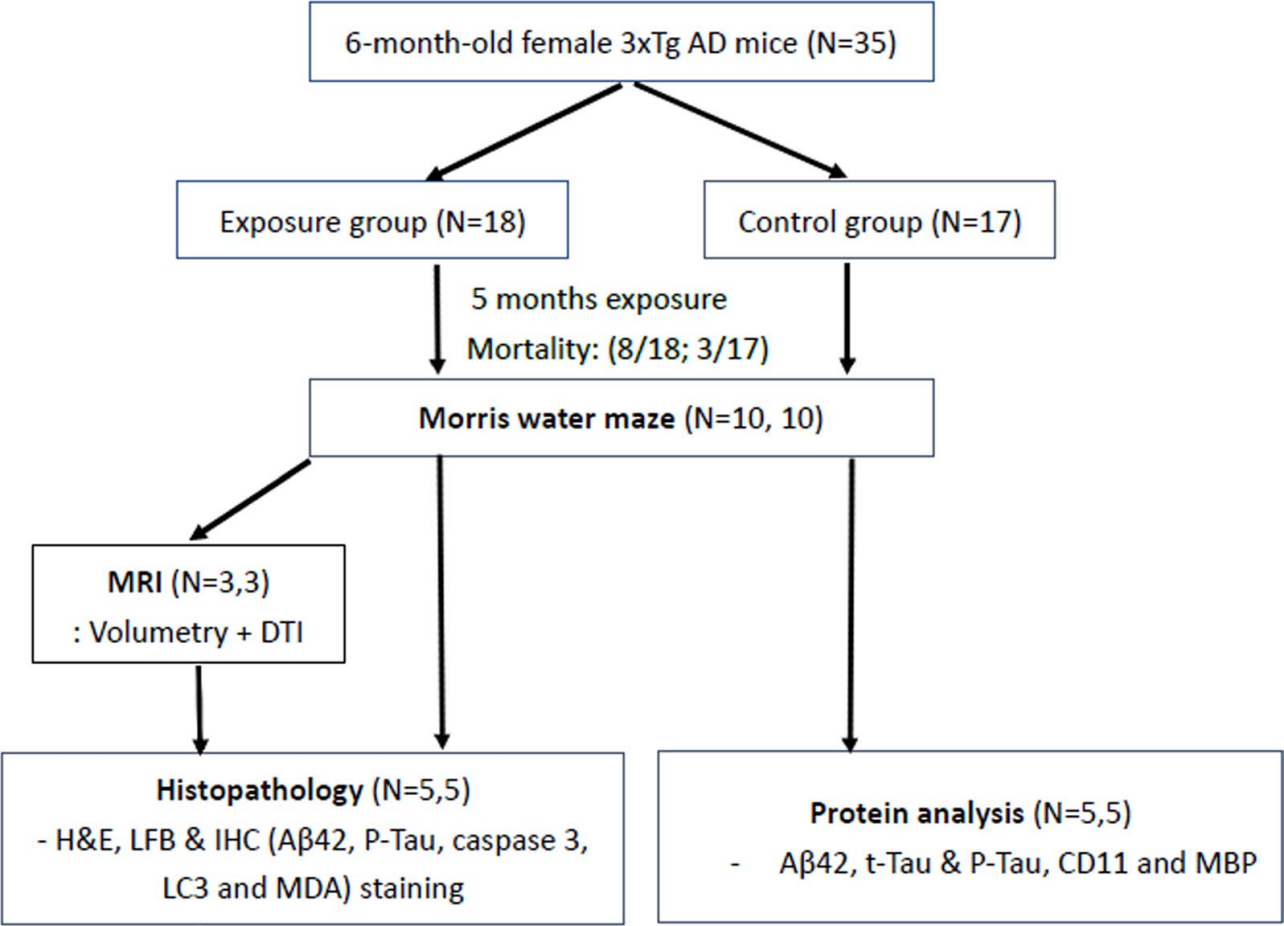
Study protocol

Thirty-five six month-old female 3xTg-AD mice were divided into exposure (n = 18) and control groups (n = 17) and were exposed to ambient or filtered air for five months during the winter of 2018 to the spring of 2019. After exposure, the surviving mice (equal number in 2 groups) were subjected to the Morris water maze test and were then sacrificed after CO_2_ anesthetization. Half of the mice were pretreated with Bouin’s solution, and then the whole brain was dissected and stored in 10% formalin for brain magnetic resonance imaging and histochemistry staining. The other half of the mice were decapitated directly, and the dissected brain was stored at -80 °C for further protein analysis. The complete experimental protocol is shown in Fig. 1.

### Morris water maze (MWM)

This standard method to evaluate the spatial learning ability of mice or rats was performed in an animal laboratory setting according to the original design [36]. The maze (100 cm diameter pool) was filled with water to a depth of 30 cm and maintained at 24 ± 1 °C with a thermoregulator. A platform 1 cm below the water surface was placed in one quadrant of the maze, and 4 markers as cues were attached to the wall of each quadrant. In the first 4 days of the experiment (the acquisition trial), each mouse was trained to find the platform within 60 s from each quadrant of the maze three times. If the mouse failed, it was placed directly on the platform for an additional 10 s. On the fifth day (the probe trial), the mouse was forced to swim without platforms in the pool for 60 s. The escape latency and related parameters were recorded with an animal behavior/trajectory tracking analysis system (Noldus EthoVision 3.1, The Netherlands).

### Magnetic resonance image (MRI)

Three mice in each group were selected for ex vivo MRI brain image analysis. Mice were anesthetized and perfused with Bouin’s solution to fix the brain tissue in advance. Whole brains were dissected and stored in 10% formalin. Brain image acquisition was performed on a 3 T MRI system (Achieva, Philips Health care, Best, Netherlands) with an ultrahigh-strength gradient coil of 675 mT/m in maximal intensity. Scheduled brain MRI scan sequencing was available for every sample, including high-resolution axial T2-weighted imaging (TR/TE = 4000/34, slice thickness = 0.5 mm, slice number = 35), for volumetric analysis of the region of interest [37]. Diffusion tensor imaging (DTI) was performed with the following parameters: TR = 2000 ms, TE = 37 ms, difference between diffusion gradient pair (Δ) = 20 ms, diffusion gradient pulse duration (δ) = 4.5 ms, diffusion gradient strength = 19.87 G/cm, and b-values = 1000 s/mm^2^. The image data had a slice thickness of 0.5 mm, a total slice number of 30, a field of view (fov) of 35 cm × 35 cm, and a matrix of 192 × 192. Data analysis was performed using ImageJ software [38]. The eigenvalues (λ1, λ2, and λ3) derived from the diffusion tensor were used to calculate the following DTI metrics: fractional anisotropy (FA), mean diffusivity (MD), axial diffusivity (AxD), and radial diffusivity (RD) [39]. Regions of interest (ROIs) were selected in the optic tract, anterior commissure, corpus callosum, internal capsule, external capsule and fimbria according to the mouse brain atlas. The mean DTI metric of each tract was averaged using computer software.

### Histopathological and immunohistochemistry staining

**Fig. 2:**
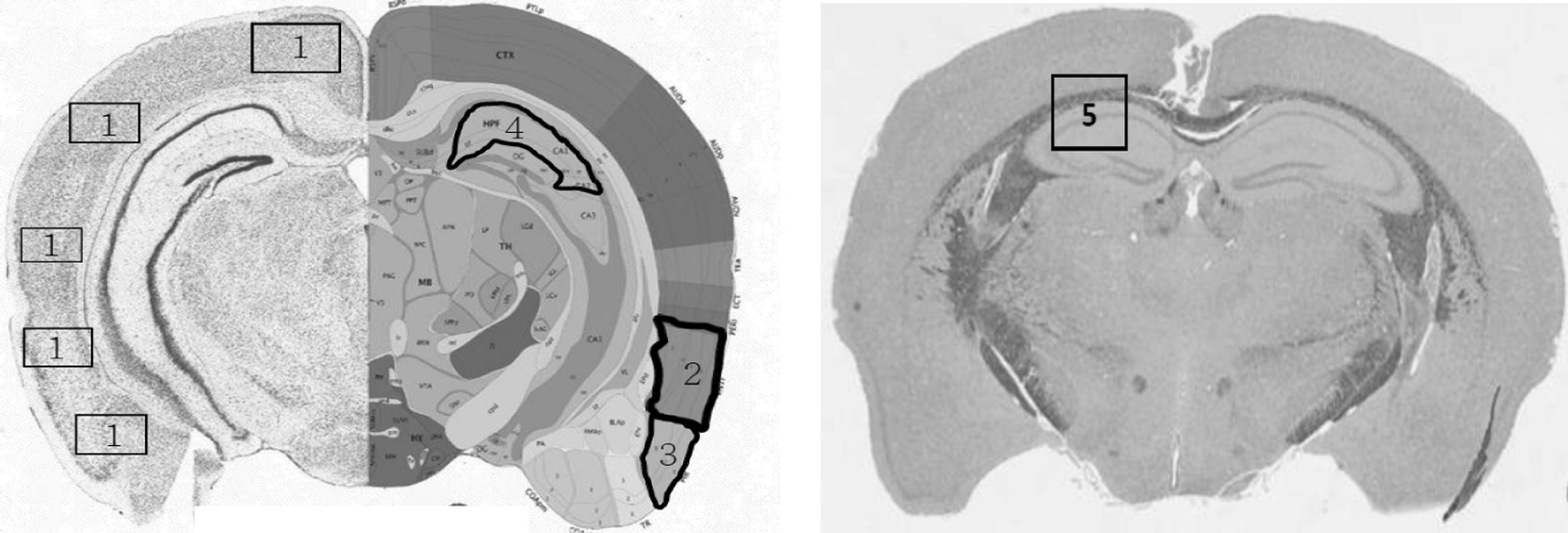
Counting of viable neuron cells and myelin density in selected regions of brain sections. The selected regions included the general cerebral cortex (1), the entorhinal cortex (2), the piriform cortex (3), and the hippocampus (4) with H&E staining and the corpus callosum (5) with LFB staining

Five mice in each group were utilized for this analysis. The formalin-fixed brains embedded in paraffin wax were sectioned coronally at a thickness of 3–5 μm. Paraffin sections were rehydrated and stained with hematoxylin and eosin (H&E) and cresyl violet (Nissl stain) to determine the viability of neurons, and myelin structure was stained with Luxol fast blue (LFB). A well-trained assistant counted the viable neurons in the selected cortical regions, including the cerebral, entorhinal, piriform, and hippocampal CA1 areas (Fig. 2). The myelin density ratio was determined in the corpus callosum on serial coronary sections of LFB-stained slides between the exposure and control groups (Fig. 2). The analyses were performed with ImageJ software as previously reported [40].

For immunohistochemistry (IHC) staining, Aβ42, P-tau, caspase 3, microtubule-associated protein 1A/1B-light chain 3 (LC3) and malondialdehyde (MDA) antigen retrievals were performed in boiling citrate buffer (pH 6.0) and incubated for 20 min. Then, the sections were blocked with the following individual primary antibodies: Aβ42 (MOAB-2, NBP2-13,075; Novus Bio, USA) at 200X dilution, phosphorylated tau (GTX26864; GeneTec, USA) at 50X dilution, Caspase-3 (bs-0081R; Bioss, USA) at 500X dilution, LC-3 (MAP LC3α/β antibody G4, Santa Cruz, USA) at 300X dilution, and MDA (ab6463; Abcam, USA) at 200X dilution. Tissue sections were then counterstained with 3,3’-diaminobenzidine (DAB) and hematoxylin.

### Western blot analysis

Another five fresh frozen mouse brains in each group were used for protein analysis. Each brain was divided into 3 parts: hippocampus, cerebral cortex and subcortical regions (defined as white matter region). Detailed western blot analysis protocols referenced previous studies with some modifications [41, 42]. Proteins were extracted from brain samples measured with a BCA Protein Assay Kit. Proteins were then electrophoresed with individual sodium dodecyl sulfate polyacrylamide gel electrophoresis (SDS–PAGE), transferred and blocked on polyvinylidene fluoride membranes. The processed membranes were incubated with the following different primary antibodies overnight at 4 °C: beta amyloid 1–42 antibody (Aβ42, ab201060; 1:1000), total-tau (t-tau, GTX112981; 1:1000) and phosphorylated-tau (P-tau, GTX24864; 1:1000), autophagosome marker (LC3B, LC2B (D11) XP@ Rabbit mAb, #3868; 1:1000), microphage and microglial marker (anti-CD11, ab133357; 1:1000), myelin basic protein (MBP, GTX100042; 1:1000), and beta actin (ab133357; 1:1000), purchased from GeneTex (Irvine, CA, USA), Abcam (Cambridge, UK), and Cell Signaling Technology (Danvers, MA, USA). After washing, the membranes were incubated with either anti-rabbit (1:10,000) or anti-mouse (1:10,000) horseradish peroxidase-labeled secondary antibodies purchased from GeneTex (Irvine, CA, USA). Enhanced chemiluminescence (ECL) was used for immunoreaction, and the imaging results were taken by the BioSpectrum 810 Imaging System (UVP, Upland, CA, USA). The protein expression levels were calculated using Image-Pro version 4 (Media Cybernetics, Inc., MD, USA).

### Statistical analyses

All statistical analyses were performed with SPSS software package version 19.0 for Windows. Data are presented as the mean ± standard error (SE). Independent Student’s t-test and the Wilcoxon rank-sum test were used for group comparisons. The level of significance was set at *P* < 0.05.

## Results

### ***Concentration and chemical composition of PM***_***2.5***_

The mean mass concentration of PM_2.5_ during the exposure period was 13.85 μg/m^3^ (range from 5.88 to 18.79), which was similar to the annual mean value of 13.0 μg/m^3^ reported by the Taipei city government in 2018. Among the chemical components of PM_2.5_, the most abundant water-soluble ions were SO_4_^2−^ (67.09%) and NH^4+^ (22.44%), whereas Ca, K, Na were the most common metal components (data not shown). It could be estimated that this ambient PM_2.5_ mostly came from traffic exhaust.

### Animal mortality and the morris water maze

There were no significant differences in the mean body weights of the exposure and control groups from the baseline to the end point (26.9–30.9 g VS. 26.8–32.9 g). However, 8 transgenic mice in the exposure group and 3 in the control group died during the experimental period; the mortality rate was obviously higher in the PM_2.5_ exposure group (44.4 VS. 17.6%).

**Fig. 3:**
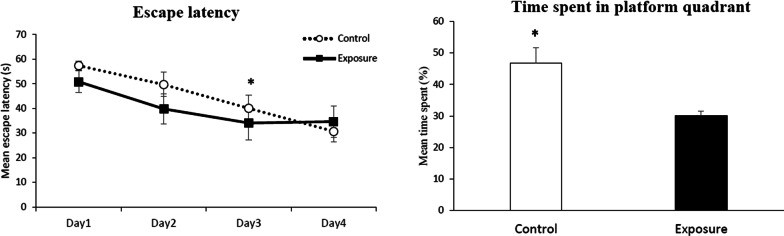
Morris water maze: acquisition and probe phase. Student T- test; *P < 0.05

In the Morris water maze test, the learning process was noted in both groups in the acquisition period (between Days 1 and 3, *P* < *0.05*, Student’s t-test; Fig. 3), but there was no significant difference between them. The exposure group showed no further learning effect on Day 4. There were also no differences in the mean swimming distance and velocity between the PM_2.5_ exposure and control groups (data not shown). In the probe stage, mice in the control group stayed in the target area for more time than those in the exposure group (46.77 ± 4.92% VS. 30.17 ± 1.40%, *P* < *0.05*, Student T-test; Fig. 3).

### Neuron viability and myelin density

**Fig. 4:**
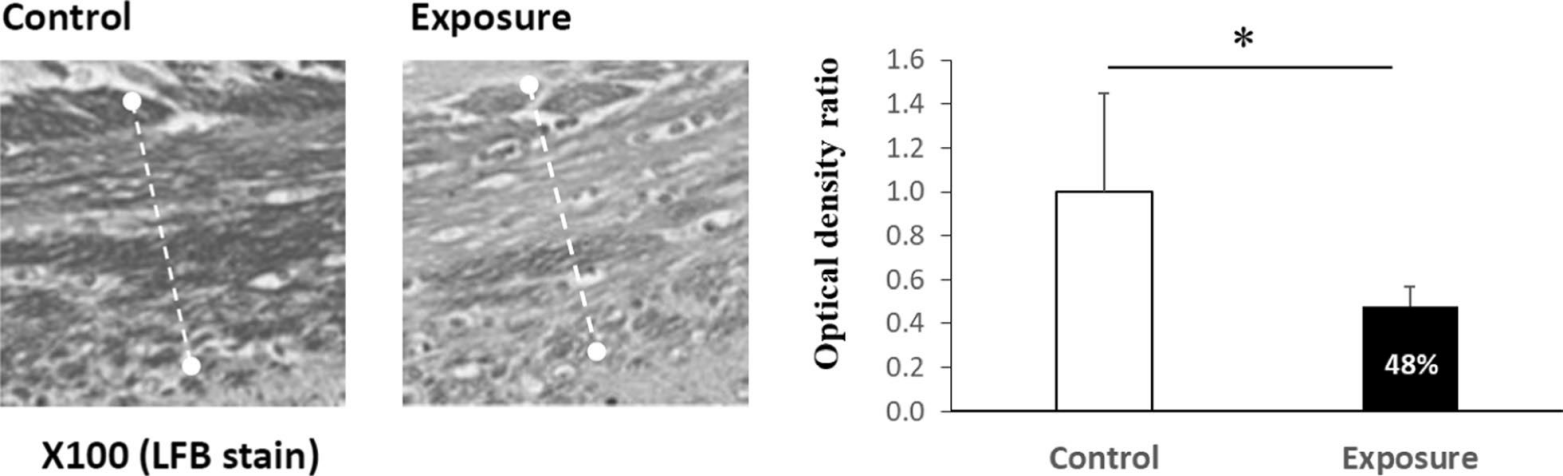
Myelin morphology and density ratio in the corpus callosum. The white dotted lines define the margin of the corpus callosum. Wilcoxon rank sum test, *P < 0.05

The viability of neurons in each selected cortex area on H&E- and Nissl-stained sections was compared between the exposure and control groups (Fig. 2). There were significant levels of neuronal loss in the cerebral and entorhinal cortex areas but not in the piriform cortex or hippocampal CA1 regions of the exposed mice (Table 1). From the LFB staining, a significant decrease in myelin density was also observed in the corpus callosum of the exposure group compared with the control group (Fig. 4).

**Table 1:** Viable neuron cells in selected regions on brain sections with H&E staining

Brain region^@^	Control	Exposure	*p* value
Cerebral cortex	61.84 ± 9.35^#^	46.24 ± 2.54	0.02*
Entorhinal cortex	15.67 ± 2.56	8.73 ± 3.92	0.04*
Piriform cortex	10.11 ± 1.89	8.44 ± 1.03	0.40
Hippocampus (CA1)	147.17 ± 22.52	124.19 ± 27.65	0.22

^**@**^Selected brain regions were located in Fig. 2

^**#**^Mean ± SD; Wilcoxon rank-sum test, *P < 0.05

### Magnetic resonance imaging

Total brain volume and specific regions of interest were located, and the volume was calculated using ImageJ software. There was significantly decreased total brain volume in the exposure group compared with the control group, but no difference was noted in the corpus callosum (Table 2). Some specific areas, such as the piriform cortex, entorhinal cortex and CA1 of the hippocampus, were also located on specific MRI sections. We did not find a significant difference between the exposure and control groups (data not shown).

**Table 2:** Calculated volumes of brain areas from the MRI study

Area/(mm^3^)	Control	Exposure	*p* value
Total cerebrum	490.24 ± 13.93^**#**^	462.15 ± 5.69	0.03*
Corpus callosum	11.42 ± 0.69	10.51 ± 1.04	0.28

^#^Mean ± SD; Wilcoxon rank-sum test, *P < 0.05

Diffusion tensor imaging (DTI) technology in magnetic resonance imaging (MRI) has been widely used for the in vivo assessment of white matter integrity. In normal white matter, axons are regularly aligned and well myelinated; the random diffusion of water molecules is restricted to a predominant orientation [43]. Fractional anisotropy (FA) presents this preference and is used to assess tract integrity but without specificity of pathology [44]. Mean diffusivity (MD) is an inverse measure of membrane density and is sensitive to changes in cellularity, edema and necrosis. Intact white matter tissue should have high FA and low MD. To evaluate the pathological characteristics, axial diffusivity (AxD) reveals the rate of water diffusion along the longitudinal axis and is associated with axonal degeneration [45, 46]. Radial diffusivity (RD) demonstrates the rate of water diffusion along the perpendicular axes and has been linked to demyelination or dysmyelination [45, 47].

**Fig. 5:**
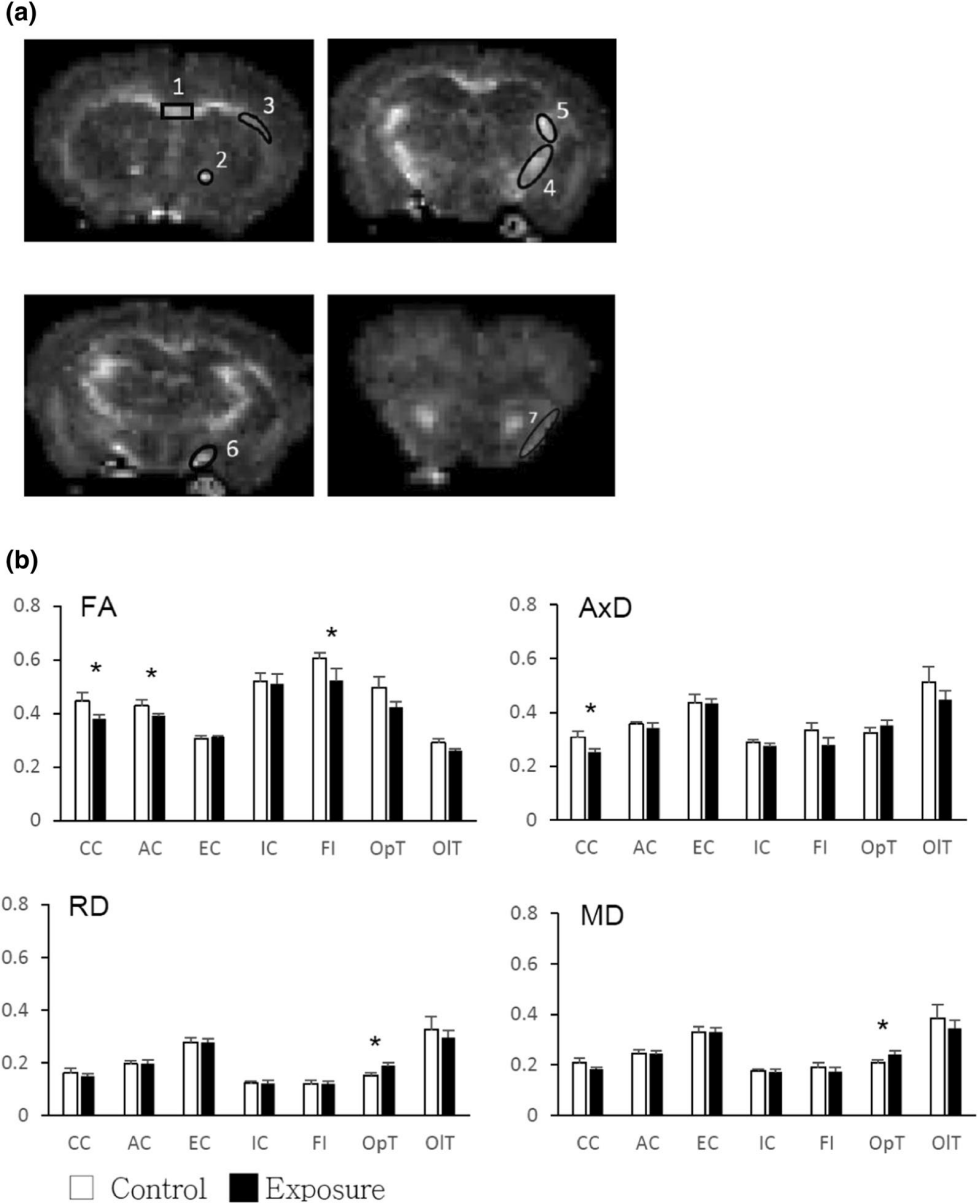
**a** Selected regions of interest (ROIs) in the MRI study. 1: corpus callosum, 2: anterior commissure, 3: external capsule, 4: internal capsule, 5: fimbria, 6: optic tract, 7: olfactory tract. **b**. DTI scalars in different tracts (ROIs) of mouse brains. Abbreviations of the DTI scalars: AxD = Axial Diffusivity, FA = Fractional Anisotropy, MD = Mean Diffusivity, and RD = Radial Diffusivity; Abbreviations of the ROIs: AC = anterior commissure, CC = corpus callosum, EC = external capsule, FI = fimbria, IC = internal capsule, OlT = olfactory tract, OpT = optic tract. Wilcoxon rank-sum test, *P < 0.05

For the DTI analysis, the selected regions of interest (ROIs) are shown in Fig. 5a. The mean FA scales were significantly decreased in most selected tracts of the exposure group, except the internal and external capsules; however, significant differences in MD and RD measurements were only observed in the optic tract (Fig. 5b). Obviously decreased AxD values were also noted in the corpus callosum of the exposure group compared with those in the control group (Fig. 5b).

### Immunohistochemistry staining

**Fig. 6:**
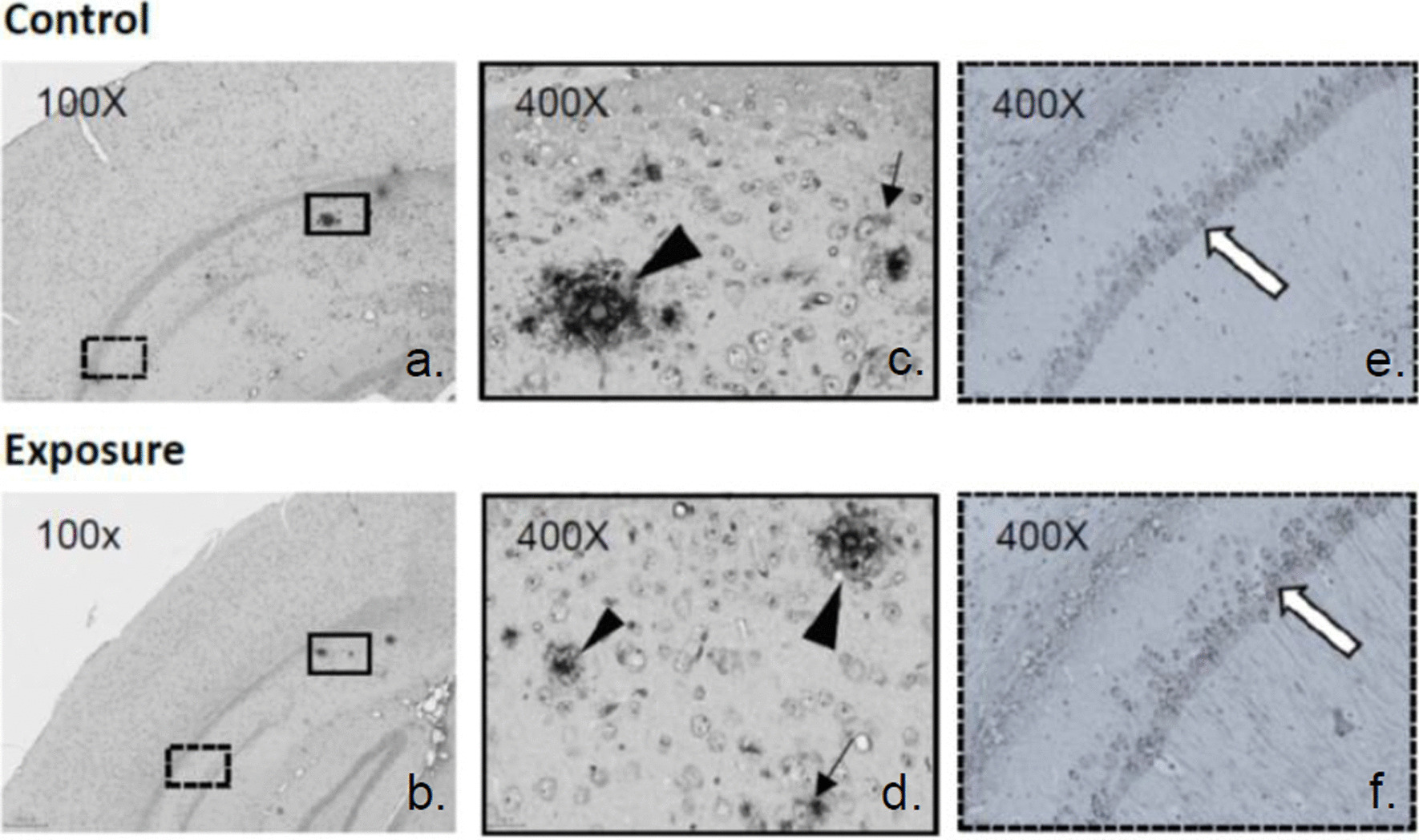
Immunohistochemistry staining of amyloid plaque, intracellular amyloid and P-tau in the hippocampus of mice

We counted the numbers of amyloid plaques and the proportion of cells with positive target staining in selected areas on different cortex parts of the transgenic mouse brain. The same coronal section was chosen to reveal the hippocampal CA1 and external cortex areas in each mouse as shown in Fig. 2, and analysed with 400 times magnification under light microscope. There was a slight increase in extracellular amyloid plaques in the hippocampus and the ratio of neurons with intracellular amyloid in other parts of the cortex of the exposed mouse brain, but the difference was not significant compared with the control group (Fig. 6a, b, c & d; Addittional file 1: Table S1). This tendency was also observed in neurons with P-tau pathology in the hippocampus of exposed mice but not in other cortical parts (Fig. 6e & f). There were also no differences in the expression of caspase-3, LC-3 and MDA between the two groups.

Subfigures c & d are enlarged images from the close tangles in subfigures a & b to reveal the dense amyloid plaques (black triangles) and the intracellular diffuse amyloid (thin arrows); subfigures e & f are enlarged images from the dotted tangles in subfigures a & b (similar position with different staining) to reveal the intracellular P-tau (white arrows).

### Protein analysis

**Fig. 7:**
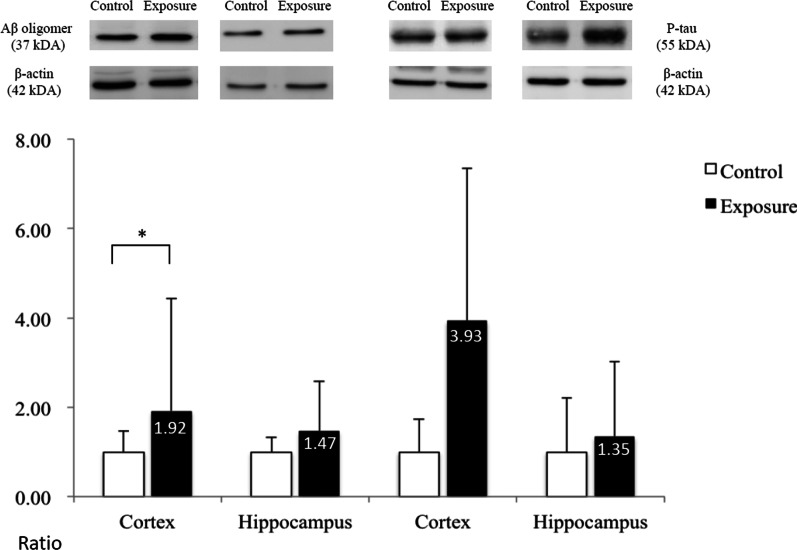
Protein expression in the cortex of mouse brains. The ratio of expression of Aβ oligomer and P-tau in the cortex and hippocampus between the exposure and control groups; Wilcoxon rank-sum test, *P < 0.05

**Fig. 8:**
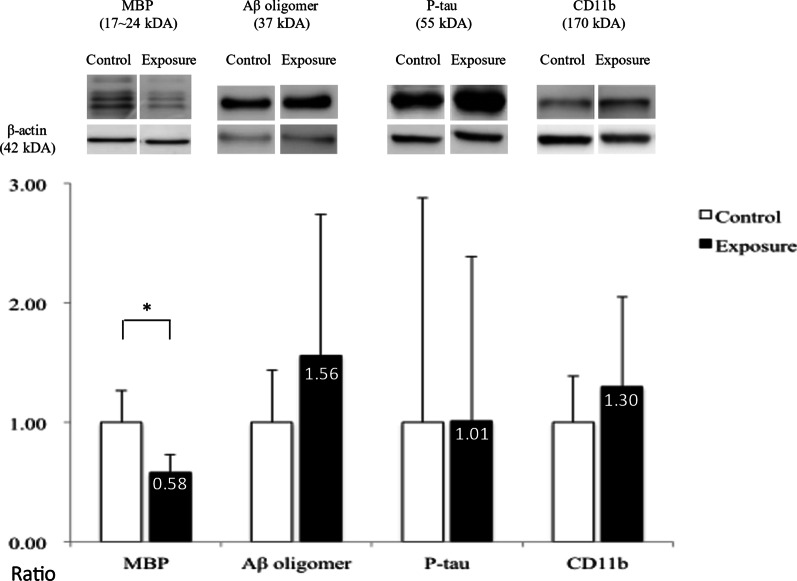
Protein expression in the white matter of mouse brains. The ratios of expression of MBP, Aβ oligomer, P-tau and CD11b in the white matter between the exposure and control groups; Wilcoxon rank-sum test, *P < 0.05

The amounts of target proteins were compared separately in the cerebral cortex and white matter regions between the 2 groups. In the cortex part, the expression of Aβ42 oligomer was increased significantly in the whole cerebral cortex, but not in the hippocampus, of the exposure group as compared with the control group (*P* < 0.05); but with only a trend and without a significant difference appearing in P-tau, the other pathological target protein of Alzheimer disease, between the two groups (Fig. 7). In the white matter part, the MBP level was also significantly decreased in the white matter of the exposure group (*P* < 0.05; Fig. 8), as indicated by the myelin damage revealed by LFB staining. There was also no significant difference in the expression levels of the Aβ42 oligomer, P-tau or CD11b.

## Discussion

**Fig. 9:**
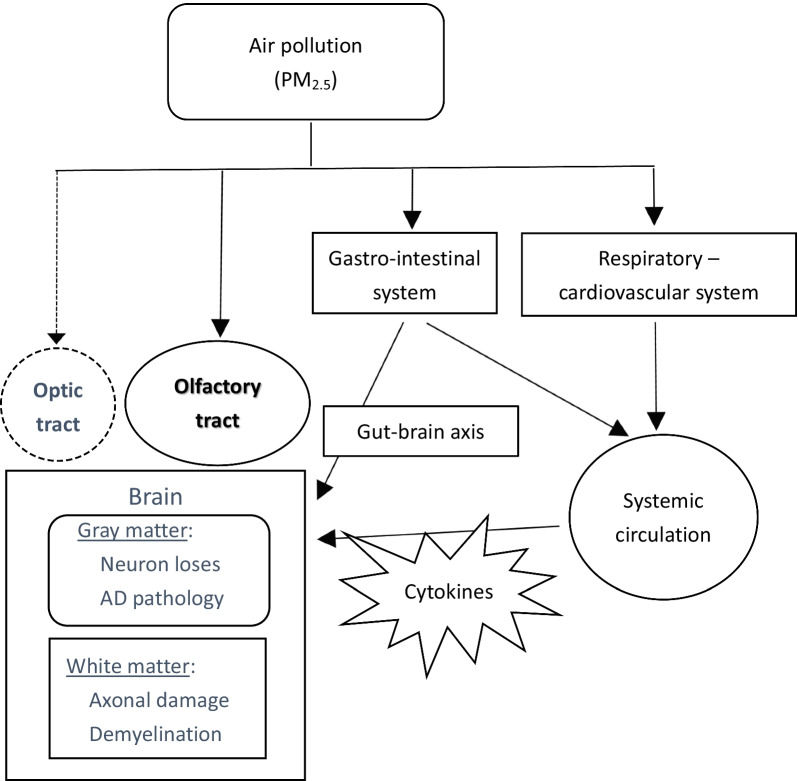
Model of the pathway and mechanism of brain damage due to exposure to PM_2.5_. Ambient PM2.5 particles in air pollution enter the human body mostly from the airway. They could penetrate deeply into the lung, irritate and corrode the alveolar wall. PM2.5 also enters the gastro-intestinal tract. Inflammation damage and oxidative stress are aroused in both pathways and toxic agents, such as cytokines are released into the systemic circulation. PM2.5 and related toxic agents would invade and damage the central nervous system (brain) via the systemic circulation, olfactory tract, direct gun-brain axis, and even the optic tract. Solid line presents the proved pathways and dotted line means the suggested new one

PM_2.5_, due to the small size of particles that can penetrate deeply into the respiratory tract and trigger various health problems, is widely considered to be the most harmful agent among the components of air pollutants [48]. In addition to the well-known respiratory and cardiovascular diseases, PM_2.5_ also causes brain insults, including a variety of neurodegenerative diseases, especially AD [49]. PM_2.5_ invades the brain directly via the olfactory nerve and can also destroy the integrity of the blood–brain barrier (BBB) via the systemic circulation [50, 51]. Peripheral systemic inflammatory materials cross the BBB and easily reach the brain, and microglia are also activated to induce neuroinflammation [49, 52]. More proinflammatory cytokines, such as IL-1β and TNF-α, are released and then cause the degeneration of neurons and axons [53]. In addition, PM_2.5_ could enter the gastrointestinal (GI) tract directly and indirectly to cause imbalances in the intestinal microecology. The malfunctional gut-brain axis has been shown to have strong relationships with the development or exacerbation of many neurodegenerative diseases (Fig. 9, solid line) [54–56]. Epigenetic effects of PM_2.5_ have also been reported, such as direct genomic damage by reactive oxidation or changes in DNA methylation in disease-related genes [49, 57–59].

Except for the general routes of PM_2.5_ neurotoxicity described above, the exact pathological mechanism associated with dementia and AD still needs further clarification. From epidemiological studies, most results revealed a negative association of cognition with PM_2.5_ concentration [60–62]. In this study, with chronic exposure to low-dose PM_2.5_, there was no significant difference in spatial learning function, but memory easily decayed after exposure, as shown by the Morris water maze test. Previous similar animal studies with prominent memory insult might be dose-dependent [63, 64]. Relatively low-dosage exposure, similar to the experimental design of the ambient atmosphere environment in this study, only induced minimal cognitive or behavioral changes. A similar finding was also reported in our previous study with a rodent model [18].

Therefore, certain brain damage and body insult still existed after chronic exposure to ambient air pollution. Even though there was no significant reduction in body weight gain during the 5 month period, the mortality rate of exposed mice was 2.5 times higher than that of the control mice. The same tendency was also shown in our previous study [32]. From the brain MRI measurements, the total brain volume of exposed mice was significantly decreased compared with that of the control group, but this difference was not detected in different brain cortical regions, including the hippocampus. Under microscopic examination, neuronal loss was more obvious in the general cerebral and entorhinal cortex areas and was not observed in the hippocampal area. For the sparing of the hippocampus in volumetric atrophy and neuron loss, these findings could explain the negative result of spatial learning dysfunction in the water maze and may also imply that the minimal brain insult caused by a low dose of PM_2.5_ was not directly due to Alzheimer’s-related pathology.

This phenomenon was also supported by the measurements of the pathognomonic markers of AD. From the IHC staining results, there were only tendencies of increase in extracellular plaques or intracellular amyloid and P-tau in the cortex after exposure. Western blot analysis showed a significant increase in Aβ42 production in the cerebral cortex of the exposed mouse brain. This result seemed to conflict with previous similar animal studies, which reported elevated levels of Aβ and P-tau pathology in the brain after exposure [17, 24, 65]. However, most studies designed exposure to higher levels of PM (ranging from 65.7 to 468.0 μg/m^3^), representing several times the human exposure in the real world [17, 23, 53]. Therefore, we postulated the possibility that chronic exposure to ambient air pollution with a mean PM_2.5_ concentration of 13.85 μg/m3 in this 3xTg-AD mouse model was able to increase the production of toxic amyloid and P-tau but was not severe enough to induce subsequent and typical cascade damage in Alzheimer’s disease, as reported previously. However, aging effects and longer exposure durations were not validated in this experiment.

Many neuroimaging studies have reported associations between exposure to air pollution and total brain volume reduction in children and elderly individuals; however, these results were variable when studies focused on gray matter, especially subcortical brain structure volumes (i.e., hippocampus, amygdala, and basal ganglia) [60, 66, 67]. In contrast, more consistent patterns of air pollution-related reduction in total and different associated areas (i.e., frontal, parietal, temporal and corpus callosum) of white matter volumes were reported [60, 66–68]. Recently, white matter demyelination was also reported in a mouse model with PM_2.5_ exposure and was thought to be induced by promoting neuroinflammation or impairing myelin repair [69]. From the results of our study, decreased myelination of the corpus callosum and reduced MBP expression in the white matter of mice exposed to PM_2.5_ also support this point of view. However, there was no significantly increased expression of CD11b at the same time. White matter damage by microglia-induced neuroinflammation may not be the main and only cause.

From the DTI measurements in this study, impaired integrity of white matter tracts was observed in most selected tracts of exposed mice, except for the internal and external capsules, which have also been less reported in AD pathology. However, damage to the axonal membrane was only observed in the optic tract. This ambient air pollution may not have caused severe damage to the white matter, a finding that also corresponded with the other pathological findings mentioned above. Most interestingly, the optic tract has seldom been reported to be affected by air pollution. In one animal experiment, infant mice exposed to environmental tobacco smoking showed decreased myelination of the optic nerve fiber [70]. Our study also proved demyelinating damage in the optic tract (increased RD value). Therefore, in addition to the well-known direct pathway of air pollutants into the brain via the olfactory nerve, the optic nerve and tract system may be another alternative route. Unfortunately, the tissues of eyeball and optic nerves were not preserved for further prove. Activation of microglia and macrophages to cause neuroinflammation is thought to play a major role in pollutant-related white matter lesions. However, the marker of neuroinflammation (CD11b) was not significantly elevated in subcortical white matter regions after exposure. Iba-1, another common microglia-related marker, was found with upregulated expression only in cerebral cortex after ambient exposure of 3 months in our previous study [32]. There was also suspected axonal damage in the corpus callosum from DTS study. Both of these findings suggest that it is not just a unique pathological mechanism that is involved in white matter insult by air pollution; and the PM_2.5_ neurotoxicity may be different in different brain regions. Further studies may be needed to clarify these results, especially to include complete inflammatory surveys and also vascular factors.

There were some shortcomings in this study design. First, the mortality rate of AD transgenic mice with longer exposure times was very high, and the sample size was not sufficient, especially to study gray and white matter together at the same time. Second, we tried to investigate the possible mechanism of neurotoxicity induced by air pollution as much as possible and could not see through the interactive pathway. Third, the 6 month-old transgenic mice coincided with middle age in humans. Previous epidemiological studies on air pollution-induced neurotoxicity have mostly been reported in children and elderly individuals. Therefore, younger or elderly mice may be better choices in this model.

## Conclusion

In conclusion, this pilot study investigated the white matter insult of AD transgenic mice with chronic ambient air pollution. The PM_2.5_ concentration during the 5 months of exposure mostly did not exceed the WHO criteria. However, it still caused some brain damage, such as gross brain atrophy, cortical neuron damage and multiple white tract damage, even at this lower toxic concentration. After exposure, the typical amyloid cascade pathology did not appear prominently in the vulnerable brain region, and memory function was also not severely affected. Except the cortical neuron lose, white matter damage was also noted in several subcortical regions, including optic tract. These results imply that multiple pathogenic pathways induce brain injury by air pollution, and the optic nerve is possibly another direct route of air pollution invasion in addition to the olfactory nerve (Fig. 9, dotted line).

## Supplementary Information

**Additional file 1**. **Table S1**. The ratio of neurons with diffuse Aβ_42_ in cerebral, entorhinal and piriform cortex and Aβ_42_ plaques in hippocampal CA1 area from immunohistochemistry study.

## Data Availability

Not applicable.

## References

[1] Choi J, Oh JY, Lee YS, Min KH, Hur GY, Lee SY, et al. Harmful impact of air pollution on severe acute exacerbation of chronic obstructive pulmonary disease: particulate matter is hazardous. Int J Chron Obstruct Pulmon Dis. 2018;13:1053–9. 10.2147/COPD.S156617. https://pubmed.ncbi.nlm.nih.gov/29681728https://www.ncbi.nlm.nih.gov/pmc/articles/PMC5881527/. 10.2147/COPD.S156617 PMC5881527 29681728

[2] Cohen AJ, Brauer M, Burnett R, Anderson HR, Frostad J, Estep K, et al. Estimates and 25-year trends of the global burden of disease attributable to ambient air pollution: an analysis of data from the global burden of diseases study 2015. The Lancet. 2017;389(10082):1907–18. 10.1016/S0140-6736(17)30505-6. https://www.sciencedirect.com/science/article/pii/S0140673617305056 10.1016/S0140-6736(17)30505-6 PMC5439030 28408086

[3] Almetwally AA, Bin-Jumah M, Allam AA (2020). Ambient air pollution and its influence on human health and welfare: an overview. Environ Sci Pollut Res.

[4] Cheng T-J, Hwang J-S, Wang P-Y, Tsai C-F, Chen C-Y, Lin S-H, et al. Effects of concentrated ambient particles on heart rate and blood pressure in pulmonary hypertensive rats. Environ Health Perspect. 2003;111(2):147–50. 10.1289/ehp.5464. https://pubmed.ncbi.nlm.nih.gov/12573896https://www.ncbi.nlm.nih.gov/pmc/articles/PMC1241341/. 10.1289/ehp.5464 PMC1241341 12573896

[5] Chuang K-J, Yan Y-H, Chiu S-Y, Cheng T-J. Long-term air pollution exposure and risk factors for cardiovascular diseases among the elderly in Taiwan. Occup Environ Med. 2011;68(1):64. 10.1136/oem.2009.052704. http://oem.bmj.com/content/68/1/64.abstract. 10.1136/oem.2009.052704 20833756

[6] Franklin BA, Brook R, Arden Pope C. Air pollution and cardiovascular disease. Curr Probl Cardiol. 2015;40(5):207–38. 10.1016/j.cpcardiol.2015.01.003. https://www.sciencedirect.com/science/article/pii/S0146280615000043. 10.1016/j.cpcardiol.2015.01.003 25882781

[7] Schraufnagel DE, Balmes JR, Cowl CT, De Matteis S, Jung S-H, Mortimer K, et al. Air pollution and noncommunicable diseases: a review by the forum of international respiratory societies’ environmental committee, part 2: air pollution and organ systems. Chest. 2019;155(2):417–26. 10.1016/j.chest.2018.10.041. https://www.sciencedirect.com/science/article/pii/S0012369218327223. 10.1016/j.chest.2018.10.041 PMC6904854 30419237

[8] Yan Y-H, Huang C-H, Chen W-J, Wu M-F, Cheng T-J. Effects of diesel exhaust particles on left ventricular function in isoproterenol-induced myocardial injury and healthy rats. Inhal Toxicol. 2008;20(2):199–203. 10.1080/08958370701861082. https://pubmed.ncbi.nlm.nih.gov/18236234. 10.1080/08958370701861082 18236234

[9] Clifford A, Lang L, Chen R, Anstey KJ, Seaton A. Exposure to air pollution and cognitive functioning across the life course – a systematic literature review. Environ Res. 2016;147:383–98. 10.1016/j.envres.2016.01.018. https://www.sciencedirect.com/science/article/pii/S0013935116300172. 10.1016/j.envres.2016.01.018 26945620

[10] Peters R, Peters J, Booth A, Mudway I (2015). Is air pollution associated with increased risk of cognitive decline? a systematic review. Age Ageing.

[11] Calderón-Garcidueñas L, Avila-Ramírez J, Calderón-Garcidueñas A, González-Heredia T, Acuña-Ayala H, Chao C-k, et al. Cerebrospinal fluid biomarkers in highly exposed PM 2.5 urbanites: the risk of & nbsp; alzheimer’s and parkinson’s diseases in& nbsp; young Mexico City residents. J Alzheimers Dis. 2016;54:597–613. 10.3233/JAD-160472. 10.3233/JAD-160472 27567860

[12] Fu P, Guo X, Cheung FMH, Yung KKL. The association between PM2.5 exposure and neurological disorders: a systematic review and meta-analysis. Sci Total Environ. 2019;655:1240–8. 10.1016/j.scitotenv.2018.11.218. https://www.sciencedirect.com/science/article/pii/S0048969718345741. 10.1016/j.scitotenv.2018.11.218 30577116

[13] Hong Y-C, Lee J-T, Kim H, Kwon H-J. Air pollution: a new risk factor in ischemic stroke mortality. Stroke. 2002;33(9):2165–9. 10.1161/01.str.0000026865.52610.5b. https://pubmed.ncbi.nlm.nih.gov/12215581. 10.1161/01.str.0000026865.52610.5b 12215581

[14] Livingston G, Huntley J, Sommerlad A, Ames D, Ballard C, Banerjee S, et al. Dementia prevention, intervention, and care: 2020 report of the Lancet Commission. The Lancet. 2020;396(10248):413–46. 10.1016/S0140-6736(20)30367-6. https://www.sciencedirect.com/science/article/pii/S0140673620303676. 10.1016/S0140-6736(20)30367-6 PMC7392084 32738937

[15] Hardy J, Selkoe DJ. The amyloid hypothesis of Alzheimer & #039;s disease: progress and problems on the road to therapeutics. science. 2002;297(5580):353. 10.1126/science.1072994. http://science.sciencemag.org/content/297/5580/353.abstract. 10.1126/science.1072994 12130773

[16] Iaccarino L, La Joie R, Lesman-Segev OH, Lee E, Hanna L, Allen IE (2021). Association between ambient air pollution and amyloid positron emission tomography positivity in older adults with cognitive impairment. JAMA Neurol.

[17] Bhatt DP, Puig KL, Gorr MW, Wold LE, Combs CK. A pilot study to assess effects of long-term inhalation of airborne particulate matter on early Alzheimer-like changes in the mouse brain. PLoS One. 2015;10(5):e0127102. 10.1371/journal.pone.0127102. https://www.ncbi.nlm.nih.gov/pubmed/25992783https://www.ncbi.nlm.nih.gov/pmc/PMC4439054/. 10.1371/journal.pone.0127102 PMC4439054 25992783

[18] Chuang H-C, Chen H-C, Chai P-J, Liao H-T, Wu C-F, Chen C-L, et al. Neuropathology changed by 3- and 6-months low-level PM2.5 inhalation exposure in spontaneously hypertensive rats. Part Fibre Toxicol. 2020; 10.1186/s12989-020-00388-6. 10.1186/s12989-020-00388-6 PMC7691081 33243264

[19] Herr D, Jew K, Wong C, Kennell A, Gelein R, Chalupa D, et al. Effects of concentrated ambient ultrafine particulate matter on hallmarks of Alzheimer’s disease in the 3xTgAD mouse model. Neurotoxicology. 2021;84:172–83. 10.1016/j.neuro.2021.03.010. https://www.sciencedirect.com/science/article/pii/S0161813X21000334. 10.1016/j.neuro.2021.03.010 PMC8268836 33794265

[20] Hullmann M, Albrecht C, van Berlo D, Gerlofs-Nijland ME, Wahle T, Boots AW, et al. Diesel engine exhaust accelerates plaque formation in a mouse model of Alzheimer’s disease. Part Fibre Toxicol. 2017;14(1):35. 10.1186/s12989-017-0213-5 10.1186/s12989-017-0213-5 PMC5577845 28854940

[21] Sahu B, Mackos AR, Floden AM, Wold LE, Combs CK (2021). Particulate matter exposure exacerbates amyloid-β plaque deposition and gliosis in APP/PS1 mice. J Alzheimers Dis.

[22] Calderon-Garciduenas L, Maronpot RR, Torres-Jardon R, Henriquez-Roldan C, Schoonhoven R, Acuna-Ayala H (2003). DNA damage in nasal and brain tissues of canines exposed to air pollutants is associated with evidence of chronic brain inflammation and neurodegeneration. Toxicol Pathol.

[23] Cacciottolo M, Morgan TE, Saffari AA, Shirmohammadi F, Forman HJ, Sioutas C, et al. Traffic-related air pollutants (TRAP-PM) promote neuronal amyloidogenesis through oxidative damage to lipid rafts. Free Radic Biol Med. 2020;147:242–51. 10.1016/j.freeradbiomed.2019.12.023. http://www.sciencedirect.com/science/article/pii/S0891584919321793. 10.1016/j.freeradbiomed.2019.12.023 PMC7075030 31883973

[24] Wang B-R, Shi J-Q, Ge N-N, Ou Z, Tian Y-Y, Jiang T, et al. PM2.5 exposure aggravates oligomeric amyloid beta-induced neuronal injury and promotes NLRP3 inflammasome activation in an in vitro model of Alzheimer's disease. J Neuroinflammation. 2018;15(1):132. 10.1186/s12974-018-1178-5. https://pubmed.ncbi.nlm.nih.gov/29720213https://www.ncbi.nlm.nih.gov/pmc/articles/PMC5932821/. 10.1186/s12974-018-1178-5 PMC5932821 29720213

[25] Graff-Radford J, Arenaza-Urquijo EM, Knopman DS, Schwarz CG, Brown RD, Rabinstein AA (2019). White matter hyperintensities: relationship to amyloid and tau burden. Brain.

[26] Nasrabady SE, Rizvi B, Goldman JE, Brickman AM. White matter changes in Alzheimer's disease: a focus on myelin and oligodendrocytes. Acta neuropathologica communications. 2018;6(1):22. 10.1186/s40478-018-0515-3. https://pubmed.ncbi.nlm.nih.gov/29499767https://www.ncbi.nlm.nih.gov/pmc/articles/PMC5834839/. 10.1186/s40478-018-0515-3 PMC5834839 29499767

[27] Roseborough A, Ramirez J, Black SE, Edwards JD (2017). Associations between amyloid β and white matter hyperintensities: a systematic review. Alzheimer’s Dement..

[28] Calderón-Garcidueñas L, Mora-Tiscareño A, Gómez-Garza G, Carrasco-Portugal MDC, Pérez-Guillé B, Flores-Murrieta FJ (2009). Effects of a cyclooxygenase-2 preferential inhibitor in young healthy dogs exposed to air pollution: a pilot study. Toxicol Pathol.

[29] Calderón-Garcidueñas L, Mora-Tiscareño A, Styner M, Gómez-garza G, Zhu H, Torres-Jardón R (2012). White matter hyperintensities, systemic inflammation, brain growth, and cognitive functions in children exposed to air pollution. J Alzheimers Dis.

[30] Jew K, Herr D, Wong C, Kennell A, Morris-Schaffer K, Oberdörster G, et al. Selective memory and behavioral alterations after ambient ultrafine particulate matter exposure in aged 3xTgAD Alzheimer’s disease mice. Part Fibre Toxicol. 2019;16(1):45. 10.1186/s12989-019-0323-3 10.1186/s12989-019-0323-3 PMC6878709 31771615

[31] Woodward NC, Pakbin P, Saffari A, Shirmohammadi F, Haghani A, Sioutas C, et al. Traffic-related air pollution impact on mouse brain accelerates myelin and neuritic aging changes with specificity for CA1 neurons. Neurobiol Aging. 2017;53:48–58. 10.1016/j.neurobiolaging.2017.01.007. https://pubmed.ncbi.nlm.nih.gov/28212893https://www.ncbi.nlm.nih.gov/pmc/articles/PMC5388507/. 10.1016/j.neurobiolaging.2017.01.007 PMC5388507 28212893

[32] Lee S-H, Chen Y-H, Chien C-C, Yan Y-H, Chen H-C, Chuang H-C, et al. Three month inhalation exposure to low-level PM2.5 induced brain toxicity in an Alzheimer's disease mouse model. PLoS One. 2021;16(8):e0254587. 10.1371/journal.pone.0254587. https://pubmed.ncbi.nlm.nih.gov/34437570https://www.ncbi.nlm.nih.gov/pmc/articles/PMC8389369/. 10.1371/journal.pone.0254587 PMC8389369 34437570

[33] Yan Y-H, C.-K. Chou C, Wang J-S, Tung C-L, Li Y-R, Lo K, et al. Subchronic effects of inhaled ambient particulate matter on glucose homeostasis and target organ damage in a type 1 diabetic rat model. Toxicol Appl Pharmacol. 2014;281(2):211–20. 10.1016/j.taap.2014.10.005. http://www.sciencedirect.com/science/article/pii/S0041008X14003664. 10.1016/j.taap.2014.10.005 25454026

[34] Hsu S-C, Liu SC, Huang Y-T, Lung S-CC, Tsai F, Tu J-Y, et al. A criterion for identifying Asian dust events based on Al concentration data collected from northern Taiwan between 2002 and early 2007. J Geophys Res Atmos. 2008;113: D18. 10.1029/2007JD009574

[35] Salvador CM, Chou CCK. Analysis of semi-volatile materials (SVM) in fine particulate matter. Atmos Environ. 2014;95:288–95. 10.1016/j.atmosenv.2014.06.046. http://www.sciencedirect.com/science/article/pii/S1352231014004920.

[36] Morris R. Developments of a water-maze procedure for studying spatial learning in the rat. J Neurosci Methods. 1984;11(1):47–60. 10.1016/0165-0270(84)90007-4. https://pubmed.ncbi.nlm.nih.gov/6471907. 10.1016/0165-0270(84)90007-4 6471907

[37] Cho K-H, Huang S-M, Choi C-H, Chen M-J, Chiang H-H, Buschbeck RP, et al. Development, integration and use of an ultra-high-strength gradient system on a human-size 3 T magnet for small animal MRI. PLoS One. 2019;14(6):e0217916. 10.1371/journal.pone.0217916 10.1371/journal.pone.0217916 PMC6546248 31158259

[38] Schneider CA, Rasband WS, Eliceiri KW. NIH Image to ImageJ: 25 years of image analysis. Nat Methods. 2012;9(7):671–5. 10.1038/nmeth.2089. https://pubmed.ncbi.nlm.nih.gov/22930834https://www.ncbi.nlm.nih.gov/pmc/articles/PMC5554542/. 10.1038/nmeth.2089 PMC5554542 22930834

[39] Le Bihan D, Mangin J-F, Poupon C, Clark CA, Pappata S, Molko N, et al. Diffusion tensor imaging: concepts and applications. J Magn Reson Imaging. 2001;13(4):534–46. 10.1002/jmri.1076. 10.1002/jmri.1076. 10.1002/jmri.1076 11276097

[40] Yamamoto Y, Ihara M, Tham C, Low RWC, Slade JY, Moss T, et al. Neuropathological correlates of temporal pole white matter hyperintensities in CADASIL. Stroke. 2009;40(6):2004–11. 10.1161/STROKEAHA.108.528299. https://pubmed.ncbi.nlm.nih.gov/19359623https://www.ncbi.nlm.nih.gov/pmc/articles/PMC2724668/. 10.1161/STROKEAHA.108.528299 PMC2724668 19359623

[41] Renart J, Reiser J, Stark GR. Transfer of proteins from gels to diazobenzyloxymethyl-paper and detection with antisera: a method for studying antibody specificity and antigen structure. Proc Natl Acad Sci USA. 1979;76(7):3116–20. 10.1073/pnas.76.7.3116. https://pubmed.ncbi.nlm.nih.gov/91164https://www.ncbi.nlm.nih.gov/pmc/articles/PMC383774/. 10.1073/pnas.76.7.3116 PMC383774 91164

[42] Shih C-H, Chen J-K, Kuo L-W, Cho K-H, Hsiao T-C, Lin Z-W, et al. Chronic pulmonary exposure to traffic-related fine particulate matter causes brain impairment in adult rats. Part Fibre Toxicol. 2018;15(1):44. 10.1186/s12989-018-0281-1. https://pubmed.ncbi.nlm.nih.gov/30413208https://www.ncbi.nlm.nih.gov/pmc/articles/PMC6234801/. 10.1186/s12989-018-0281-1 PMC6234801 30413208

[43] Chen T-F, Lin C-C, Chen Y-F, Liu H-M, Hua M-S, Huang Y-C, et al. Diffusion tensor changes in patients with amnesic mild cognitive impairment and various dementias. Psychiatry Research: Neuroimaging. 2009;173 1:15–21. 10.1016/j.pscychresns.2008.09.002. https://www.sciencedirect.com/science/article/pii/S0925492708001443. 10.1016/j.pscychresns.2008.09.002 19442496

[44] Pierpaoli C, Jezzard P, Basser PJ, Barnett A, Di Chiro G (1996). Diffusion tensor MR imaging of the human brain. Radiology.

[45] Alves GS, Oertel Knöchel V, Knöchel C, Carvalho AF, Pantel J, Engelhardt E, et al. Integrating retrogenesis theory to Alzheimer's disease pathology: insight from DTI-TBSS investigation of the white matter microstructural integrity. BioMed research international. 2015;2015:291658. 10.1155/2015/291658. https://pubmed.ncbi.nlm.nih.gov/25685779https://www.ncbi.nlm.nih.gov/pmc/articles/PMC4320890/. 10.1155/2015/291658 PMC4320890 25685779

[46] Mayo CD, Garcia-Barrera MA, Mazerolle EL, Ritchie LJ, Fisk JD, Gawryluk JR, et al. Relationship between DTI metrics and cognitive function in alzheimer’s disease. Front Aging Neurosci. 2019;10:436. 10.3389/fnagi.2018.00436. https://pubmed.ncbi.nlm.nih.gov/30687081https://www.ncbi.nlm.nih.gov/pmc/articles/PMC6333848/. 10.3389/fnagi.2018.00436 PMC6333848 30687081

[47] Bosch B, Arenaza-Urquijo EM, Rami L, Sala-Llonch R, Junqué C, Solé-Padullés C, et al. Multiple DTI index analysis in normal aging, amnestic MCI and AD. Relationship with neuropsychological performance. Neurobiol Aging. 2012;33(1):61–74. 10.1016/j.neurobiolaging.2010.02.004. https://www.sciencedirect.com/science/article/pii/S0197458010000825. 10.1016/j.neurobiolaging.2010.02.004 20371138

[48] Choi H, Kim SH. Air pollution and dementia. Dement Neurocognit Dis. 2019;18(4):109–12. 10.12779/dnd.2019.18.4.109. https://pubmed.ncbi.nlm.nih.gov/31942169https://www.ncbi.nlm.nih.gov/pmc/articles/PMC6946615/. 10.12779/dnd.2019.18.4.109 PMC6946615 31942169

[49] Shou Y, Huang Y, Zhu X, Liu C, Hu Y, Wang H. A review of the possible associations between ambient PM2.5 exposures and the development of Alzheimer's disease. Ecotoxicol Environ Saf. 2019;174:344–52. 10.1016/j.ecoenv.2019.02.086. https://www.sciencedirect.com/science/article/pii/S0147651319302519. 10.1016/j.ecoenv.2019.02.086 30849654

[50] Calderón-Garcidueñas L, Franco-Lira M, Henríquez-Roldán C, Osnaya N, González-Maciel A, Reynoso-Robles R, et al. Urban air pollution: influences on olfactory function and pathology in exposed children and young adults. Exp Toxicol Pathol Off J Gesellschaft fur Toxikologische Pathol. 2010;62(1):91–102. 10.1016/j.etp.2009.02.117. https://pubmed.ncbi.nlm.nih.gov/19297138https://www.ncbi.nlm.nih.gov/pmc/articles/PMC2832203/. 10.1016/j.etp.2009.02.117 PMC2832203 19297138

[51] Elder A, Gelein R, Silva V, Feikert T, Opanashuk L, Carter J, et al. Translocation of inhaled ultrafine manganese oxide particles to the central nervous system. Environ Health Perspect. 2006;114(8):1172–8. 10.1289/ehp.9030. https://pubmed.ncbi.nlm.nih.gov/16882521https://www.ncbi.nlm.nih.gov/pmc/articles/PMC1552007/. 10.1289/ehp.9030 PMC1552007 16882521

[52] Xu M-X, Zhu Y-F, Chang H-F, Liang Y. Nanoceria restrains PM2.5-induced metabolic disorder and hypothalamus inflammation by inhibition of astrocytes activation related NF-κB pathway in Nrf2 deficient mice. Free Radic Biol Med. 2016;99:259–72. 10.1016/j.freeradbiomed.2016.08.021. https://www.sciencedirect.com/science/article/pii/S0891584916304026. 10.1016/j.freeradbiomed.2016.08.021 27554971

[53] Cheng H, Saffari A, Sioutas C, Forman HJ, Morgan TE, Finch CE (2016). Nanoscale particulate matter from urban traffic rapidly induces oxidative stress and inflammation in olfactory epithelium with concomitant effects on brain. Environ Health Perspect.

[54] Benakis C, Martin-Gallausiaux C, Trezzi J-P, Melton P, Liesz A, Wilmes P. The microbiome-gut-brain axis in acute and chronic brain diseases. Curr Opin Neurobiol. 2020;61:1–9. 10.1016/j.conb.2019.11.009. https://www.sciencedirect.com/science/article/pii/S0959438819301084. 10.1016/j.conb.2019.11.009 31812830

[55] Kish L, Hotte N, Kaplan GG, Vincent R, Tso R, Gänzle M, et al. Environmental particulate matter induces murine intestinal inflammatory responses and alters the gut microbiome. PLoS One. 2013;8(4):e62220. 10.1371/journal.pone.0062220. https://pubmed.ncbi.nlm.nih.gov/23638009https://www.ncbi.nlm.nih.gov/pmc/articles/PMC3634745/. 10.1371/journal.pone.0062220 PMC3634745 23638009

[56] Klingelhoefer L, Reichmann H (2015). Pathogenesis of Parkinson disease—the gut–brain axis and environmental factors. Nat Rev Neurol.

[57] Baccarelli A, Wright RO, Bollati V, Tarantini L, Litonjua AA, Suh HH, et al. Rapid DNA methylation changes after exposure to traffic particles. Am J Respir Crit Care Med. 2009;179(7):572–8. 10.1164/rccm.200807-1097OC. https://pubmed.ncbi.nlm.nih.gov/19136372https://www.ncbi.nlm.nih.gov/pmc/articles/PMC2720123/. 10.1164/rccm.200807-1097OC PMC2720123 19136372

[58] Tarantini L, Bonzini M, Apostoli P, Pegoraro V, Bollati V, Marinelli B, et al. Effects of particulate matter on genomic DNA methylation content and iNOS promoter methylation. Environ Health Perspect. 2009;117(2):217–22. 10.1289/ehp.11898. https://pubmed.ncbi.nlm.nih.gov/19270791https://www.ncbi.nlm.nih.gov/pmc/articles/PMC2649223/. 10.1289/ehp.11898 PMC2649223 19270791

[59] Vattanasit U, Navasumrit P, Khadka MB, Kanitwithayanun J, Promvijit J, Autrup H, et al. Oxidative DNA damage and inflammatory responses in cultured human cells and in humans exposed to traffic-related particles. Int J Hyg Environ Health. 2014;217(1):23–33. 10.1016/j.ijheh.2013.03.002. https://www.sciencedirect.com/science/article/pii/S1438463913000400. 10.1016/j.ijheh.2013.03.002 23567252

[60] Chen JC, Wang X, Wellenius GA, Serre ML, Driscoll I, Casanova R (2015). Ambient air pollution and neurotoxicity on brain structure: evidence from women’s health initiative memory study. Ann Neurol.

[61] Hedges DW, Erickson LD, Kunzelman J, Brown BL, Gale SD. Association between exposure to air pollution and hippocampal volume in adults in the UK Biobank. Neurotoxicology. 2019;74:108–20. 10.1016/j.neuro.2019.06.005. https://www.sciencedirect.com/science/article/pii/S0161813X19300531. 10.1016/j.neuro.2019.06.005 31220475

[62] Wilker EH, Preis SR, Beiser AS, Wolf PA, Au R, Kloog I (2015). Long-term exposure to fine particulate matter, residential proximity to major roads and measures of brain structure. Stroke.

[63] Ku T, Li B, Gao R, Zhang Y, Yan W, Ji X, et al. NF-κB-regulated microRNA-574-5p underlies synaptic and cognitive impairment in response to atmospheric PM(2.5) aspiration. Part Fibre Toxicol. 2017;14(1):34. 10.1186/s12989-017-0215-3. https://pubmed.ncbi.nlm.nih.gov/28851397https://www.ncbi.nlm.nih.gov/pmc/articles/PMC5575838/. 10.1186/s12989-017-0215-3 PMC5575838 28851397

[64] Liu J, Yang C, Yang J, Song X, Han W, Xie M, et al. Effects of early postnatal exposure to fine particulate matter on emotional and cognitive development and structural synaptic plasticity in immature and mature rats. Brain and Behavior. 2019;9(12):e01453. 10.1002/brb3.1453. 10.1002/brb3.1453. 10.1002/brb3.1453 PMC6908876 31709780

[65] Bolton JL, Smith SH, Huff NC, Gilmour MI, Foster WM, Auten RL, et al. Prenatal air pollution exposure induces neuroinflammation and predisposes offspring to weight gain in adulthood in a sex-specific manner. FASEB J Off Publ Fed Am Soc Exp Biol. 2012;26(11):4743–54. 10.1096/fj.12-210989. https://pubmed.ncbi.nlm.nih.gov/22815382. 10.1096/fj.12-210989 22815382

[66] Calderón-Garcidueñas L, Engle R, Mora-Tiscareño A, Styner M, Gómez-Garza G, Zhu H, et al. Exposure to severe urban air pollution influences cognitive outcomes, brain volume and systemic inflammation in clinically healthy children. Brain Cogn. 2011;77(3):345–55. 10.1016/j.bandc.2011.09.006. https://www.sciencedirect.com/science/article/pii/S0278262611001850. 10.1016/j.bandc.2011.09.006 22032805

[67] de Prado Bert P, Mercader EMH, Pujol J, Sunyer J, Mortamais M. The Effects of Air Pollution on the Brain: a Review of Studies Interfacing Environmental Epidemiology and Neuroimaging. Current environmental health reports. 2018;5(3):351–64. 10.1007/s40572-018-0209-9. https://pubmed.ncbi.nlm.nih.gov/30008171https://www.ncbi.nlm.nih.gov/pmc/articles/PMC6132565/. 10.1007/s40572-018-0209-9 PMC6132565 30008171

[68] Casanova R, Wang X, Reyes J, Akita Y, Serre ML, Vizuete W, et al. A voxel-based morphometry study reveals local brain structural alterations associated with ambient fine particles in older women. Front Hum Neurosci. 2016;10:495. 10.3389/fnhum.2016.00495. https://pubmed.ncbi.nlm.nih.gov/27790103https://www.ncbi.nlm.nih.gov/pmc/articles/PMC5061768/. 10.3389/fnhum.2016.00495 PMC5061768 27790103

[69] Parolisi R, Montarolo F, Pini A, Rovelli S, Cattaneo A, Bertolotto A, et al. Exposure to fine particulate matter (PM2.5) hampers myelin repair in a mouse model of white matter demyelination. Neurochem Int. 2021;145:104991. 10.1016/j.neuint.2021.104991. https://www.sciencedirect.com/science/article/pii/S0197018621000371. 10.1016/j.neuint.2021.104991 33587955

[70] Torres LH, Annoni R, Balestrin NT, Coleto PL, Duro SO, Garcia RCT (2015). Environmental tobacco smoke in the early postnatal period induces impairment in brain myelination. Arch Toxicol.

