# Supplementary material for: White matter pathology in alzheimer’s transgenic mice with chronic exposure to low-level ambient fine particulate matter
Source: Part Fibre Toxicol. 2022 Jun 30;19:44. doi: 10.1186/s12989-022-00485-8 (PMC9245233; doi:10.1186/s12989-022-00485-8)
Supplement: Supplementary file 1 — Additional file 1. Table S1. The ratio of neurons with diffuse Aβ42 in cerebral, entorhinal and piriform cortex and Aβ42 plaques in hippocampal CA1 area from immunohistochemistry study. [file 12989_2022_485_MOESM1_ESM.docx]

Supplementary Table. 1 The ratio of neurons with diffuse Aβ_42_ in cerebral, entorhinal and piriform cortex and Aβ_42_ plaques in hippocampal CA1 area from immunohistochemistry study

| Brain region | Control | Exposure | *p*-value |
| --- | --- | --- | --- |
| Cerebral cortex | 0.031 ± 0.017 (0.02) | 0.053 ± 0.039 (0.04) | 0.403 |
| Entorhinal cortex | 0.052 ± 0.034 (0.075) | 0.076 ± 0.035 (0.067) | 0.531 |
| Piriform cortex | 0.091 ± 0.022 (0.086) | 0.126 ± 0.083 (0.083) | 0.588 |
| Hippocampus CA1 (Plaques) | 4.5 ± 2.5 (4.5) | 5.5 ± 0 (5.5) | - |

The data were shown as Mean ± SE (Median) and compared via Wilcoxon rank sum test.
